# Sex differences for clinical presentations and co-pathologies in four-repeat tauopathies

**DOI:** 10.1186/s13293-026-00899-5

**Published:** 2026-04-03

**Authors:** Ece Bayram, Danelle J. Carter, Sana Aslam, Emily Forbes, Samantha K. Holden

**Affiliations:** Department of Neurology, University of Colorado Anschutz, Aurora, 12631 E 17th Avenue CO 80045 USA

**Keywords:** Tauopathies, Frontotemporal lobar degeneration, Sex, Progressive supranuclear palsy, Corticobasal degeneration, Argyrophilic grain disease, Alzheimer’s disease, Lewy bodies, Diagnosis

## Abstract

**Background:**

Four-repeat (4R)-tauopathies cause variable clinical profiles leading to clinical misdiagnosis. While sex differences are reported in Alzheimer’s disease (AD), Lewy body disease (LBD), and clinically-defined frontotemporal dementia (FTD), little is known in 4R-tauopathies.

**Methods:**

National Alzheimer’s Coordinating Center data were used for pathologically-defined 4R-tauopathies: progressive supranuclear palsy (PSP, *n* = 175), corticobasal degeneration (CBD, *n* = 114), argyrophilic grain disease (AGD, *n* = 230), Other-4R (*n* = 67). Sex differences for clinical presentation and co-pathologies were assessed adjusting for age and multiple comparisons.

**Results:**

Most common clinical diagnosis was PSP (41%) for PSP; unspecified FTD (36%) for CBD; AD for AGD (57%) and Other-4R groups (48%), without sex differences. Females had less cognitive decline, apathy, motor symptoms; were older at cognitive, behavioral change onset. Males were more likely to demonstrate LBD co-pathology and clinical profile.

**Conclusion:**

Both females and males have low clinical diagnostic accuracy for 4R-tauopathies. Females with 4R-tauopathies may experience less severe clinical presentations and less co-pathology.

## Abstract (plain language summary)

Four-repeat-tauopathies are a group of diseases that cause brain cells to lose function and gradually die. These brain changes can cause very different outward symptoms in different people, leading to wrong clinical diagnoses. We wanted to know if this varied based on the sex of the person with this brain condition. Using data from the National Alzheimer’s Coordinating Center, we found both females and males with these diseases were often misdiagnosed during life at similar rates. Overall, females experienced less cognitive changes, apathy, and motor changes than males, and were also older when they started experiencing cognitive and behavioral changes than males. This suggests females may experience less severe symptoms than males with four-repeat-tauopathies.

## Highlights

We compared co-pathology and clinical profiles between the sexes for people with 4R-tauopathy pathologies including progressive supranuclear palsy (PSP, *n*= 175), corticobasal degeneration (CBD, *n*= 114), argyrophilic grain disease (AGD, *n*= 230), and Other-4R (*n* = 67) groups.

Overall, females had less cognitive decline, apathy, and motor symptoms; and were older at cognitive and behavioral change onset than males.

Lewy body co-pathology was more prevalent, and association with Lewy body disease clinical profile was stronger for males.

Most common clinical diagnoses were; PSP (41%) for PSP pathology; unspecified FTD (36%) for CBD pathology; AD for AGD (57%) and Other-4R pathology groups (48%), without sex differences.

## Background

Four-repeat (4R-) tauopathies are a subtype of frontotemporal lobar degeneration (FTLD), defined by the predominant accumulation of tau protein isoforms with four microtubule-binding domains [1]. Of the currently defined 4R-tauopathy subtypes, progressive supranuclear palsy (PSP), corticobasal degeneration (CBD), argyrophilic grain disease (AGD), globular glial tauopathies (GGT), and FTLD-4R tau due to *MAPT* mutations can lead to a range of cognitive, behavioral, and motor symptoms with overlapping clinical presentations [2]. Clinical profiles associated with PSP and CBD include Richardson’s syndrome, corticobasal syndrome (CBS), parkinsonism, behavioral variant frontotemporal dementia (bvFTD), and primary progressive aphasia (PPA) [3]. AGD is associated with dementia characterized by a slow progressive memory decline and parkinsonism, leading to clinical diagnoses of Alzheimer’s disease (AD), Lewy body dementia, and PSP [4]. GGT is associated with the clinical profiles of bvFTD and motor neuron disease (MND) [3]. FTLD-4R tau due to *MAPT* mutation is associated with the clinical profiles of bvFTD, PSP, and CBS [5]. Clinically distinguishing these pathologies has limited sensitivity and specificity. In fact, as diagnostic criteria are not sensitive or specific enough to differentiate PSP or CBD pathology and these pathologies have overlapping mechanisms, risk factors, and clinical profiles, International Parkinson and Movement Disorders Society (MDS) introduced diagnostic criteria for probable 4R-tauopathy [6]. Although these criteria performed better than PSP and CBD diagnostic criteria, the sensitivity (59%) and specificity (88%) were still limited.

Phenotypic heterogeneity in 4R-tauopathies is potentially compounded by sex differences. Sex differences have been reported in FTD in clinical and genetic cohorts [7, 8], although the use of pathologically defined cohorts can lead to different outcomes considering the limited clinical diagnostic accuracy for 4R-tauopathies [9]. Whether clinical diagnostic accuracy rates differ by sex is still understudied and the limited research for 4R-tauopathies so far noted no sex differences for pathology prevalence. However, studies suggest sex differences in disease onset and progression for 4R-tauopathies [10–12]. Females with PSP may have younger age at onset with faster disease progression than males [10, 11], although this was not consistent across all studies [13, 14]. Females with GGT were six years older at symptom onset than males [15]. Co-pathologies also occur in 4R-tauopathies with potential implications for clinical profile and sex differences. Approximately 20% of people with PSP or CBD pathology had Lewy body co-pathology, ~ 26% with PSP or CBD had AD pathology, up to 16% of people with PSP and 45% of people with CBD had TAR DNA-binding protein 43 (TDP-43) pathology [9, 16–18]. AGD is common in aging and found with other proteinopathies, whereas GGT is less likely to have co-pathologies [9, 16]. Compared to males, females with AGD were more likely to have TDP-43 pathology and less likely to have PSP pathology [19]. Additionally, sex differences for tau pathology burden and its clinical correlates in neurodegenerative diseases such as AD and Lewy body diseases (LBD) [20–23] support the need to investigate sex differences in 4R-tauopathies to gain better insight for diagnostic and therapeutic advances.

To address these gaps, we investigated sex differences of 4R-tauopathy clinical profiles and neurodegenerative co-pathologies. Given the overlapping profiles, we performed analyses in the overall 4R-tauopathy cohort as well as individual 4R-tauopathy subtypes.

## Methods

### Participants

Data were from National Alzheimer’s Coordinating Center (NACC) Neuropathology and Uniform Data Set (UDS) for participants with visits between September 2005 and September 2025 from 39 past and present Alzheimer’s Disease Research Centers (ADRC) across the United States [24–27]. Data are collected by trained clinicians and trained personnel using standardized evaluation. The study was approved by the institutional review boards of participating sites and conducted in accordance with the Declaration of Helsinki. Written informed consent was obtained from participants prior to participation.

We included participants with at least one subtype of 4R-tauopathy, independent of clinical diagnosis, including PSP, CBD, AGD, or Other 4R-tauopathy (including sporadic multiple systems tauopathy, GGT, FTLD-4R tau due to *MAPT* mutations). Participants with missing data for any of these 4R-tauopathy variables were excluded. These criteria resulted in a study cohort of 253 females and 283 males from 28 ADRCs.

### Neuropathology measures

Co-pathology measures included presence of FTLD-3R tau pathology, FTLD with TDP-43 pathology, amyotrophic lateral sclerosis/motor neuron disease, other FTLD, hippocampal sclerosis, ischemic/hemorrhagic/vascular pathology; presence and severity of aging-related tau astrogliopathy (ARTAG); severity of AD pathology, Lewy body pathology, substantia nigra neuron loss, and cerebral amyloid angiopathy [24]. FTLD-3R tau pathology included Pick’s disease, other tauopathy including *MAPT* mutation 3R tauopathy, chronic traumatic encephalopathy (CTE), amyotrophic lateral sclerosis/parkinsonism-dementia, tangle dominant disease, and other 3R + 4R-tauopathy. AD pathology variables included Thal amyloid β plaque score, Braak neurofibrillary tangle stage, CERAD neocortical neuritic plaque score, and CERAD semiquantitative diffuse plaque score. Lewy body staging included Lewy body pathology in olfactory bulb, amygdala, brainstem, limbic and neocortex.

### Clinical measures

Clinical measures at last visit before death were assessed for cognitive, behavioral, and motor changes, and clinical diagnosis. Meaningful impairment was based on clinician’s best judgment following examination and interview with participant and co-participant [25, 26]. Clinical diagnosis of FTD, AD, and LBD was based on available diagnostic criteria at the time of the examination.

Cognitive features included impairment on memory, orientation, judgment, language, visuospatial function, attention/concentration, cognitive fluctuations; predominant symptom first recognized as a decline in individual’s cognition; age at cognitive decline onset. CDR^®^ Dementia Staging Instrument–Sum of Boxes (CDR–SOB) was used for dementia severity.

Behavioral features included change in behavior such as apathy, depression, anxiety, hallucinations, delusions, disinhibition, irritability, agitation, personality change, rapid eye movement sleep behavior disorder; predominant symptom first recognized as a decline in individual’s behavior; age at behavioral symptom onset. Neuropsychiatric Inventory-Questionnaire (NPI-Q) total score was used for overall behavioral symptom severity.

Motor features included gait disorder, falls, tremor, slowness; predominant symptom first recognized as a decline in individual’s motor function; age at motor symptom onset. Predominant domain (cognition, behavior or motor) first recognized as changed was noted.

### Statistical analysis

R version 4.4.1 and SPSS version 31.0 (Armonk, NY: IBM Corp) were used for statistics. Demographics were compared between sexes with Chi square and t tests. Presence and staging/severity of co-pathologies were compared between sexes with age at death as a covariate in generalized linear models (GLMs). Interaction of sex and Lewy body pathology for clinical diagnosis of LBD were assessed with GLMs including age at last visit as a covariate. Specific types of cognitive, behavioral, and motor symptoms and the predominant symptomatic domain of these three were compared between sexes with Chi square tests. Clinical diagnoses and presence, severity, and onset age of behavioral and motor symptoms were compared between sexes with GLMs including age at last visit as a covariate. Cognitive status and presence, severity, and onset age of cognitive changes were compared between sexes with age at last visit and education years as covariates in GLMs. Analyses were conducted in the overall cohort and the individual 4R-tauopathy subtypes (PSP, CBD, AGD, Other). As age significantly differed for females and males, sensitivity analyses included linear models with the interaction between sex and age at last visit for onset age and severity of symptoms that differed by sex. Alpha threshold of 0.05 was considered significant. False discovery rate correction was used for multiple comparisons and false discovery rate corrected *p* values are reported.

## Results

**Fig. 1:**
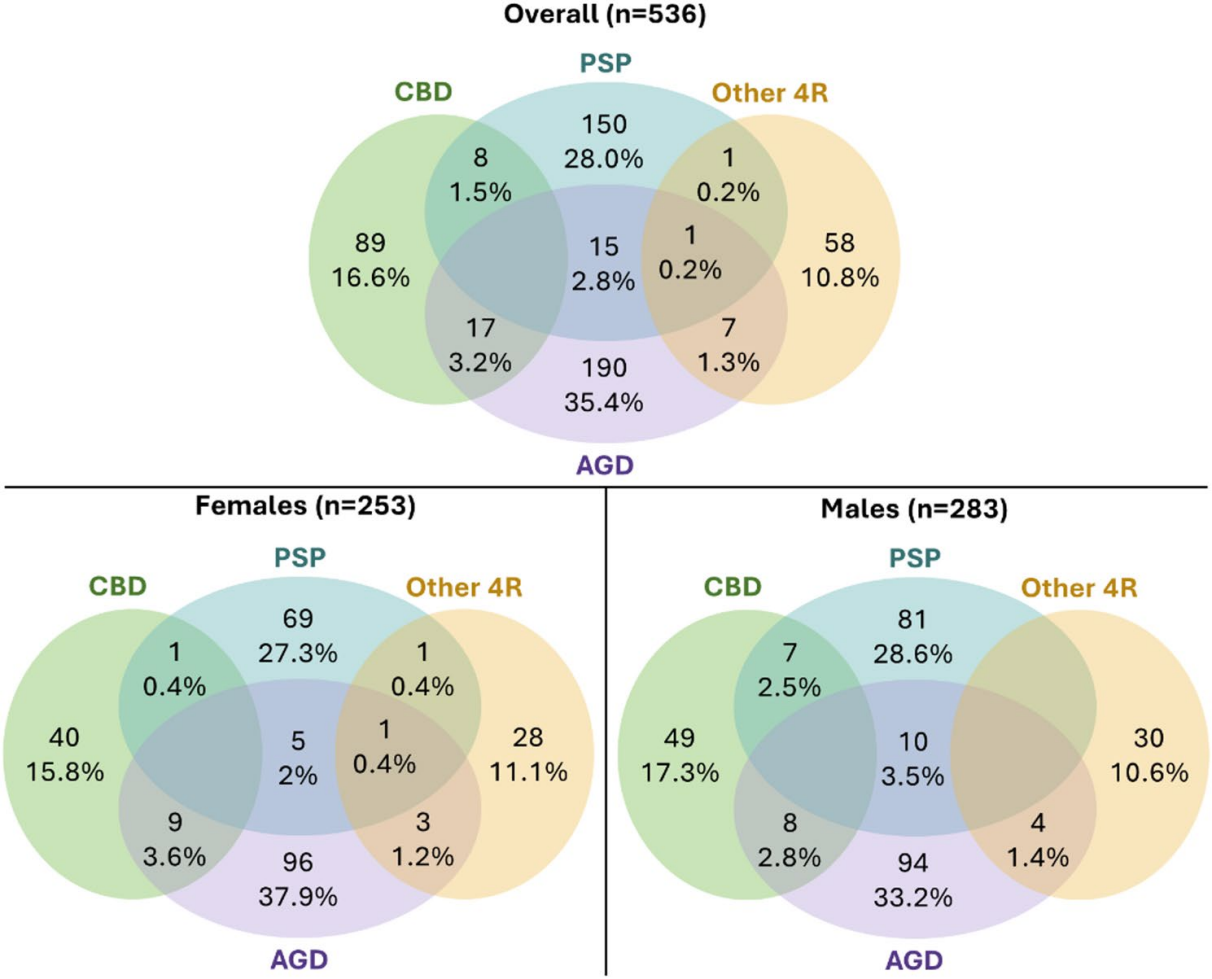
Number of participants in different 4-repeat tauopathy pathology subtypes

In the overall cohort of 253 females and 283 males, the majority (90.9%) had a single 4R-tauopathy pathological diagnosis. Overall, 175 had PSP (32.6%), 114 had CBD (21.3%), 230 had AGD (42.9%), and 67 had Other 4R-tauopathy (12.5%) with similar sex distributions by 4R-tauopathy subtype (Fig. 1).

### Sex differences for demographics (Table 1)

**Table 1:** Demographics for the overall 4R cohort and subtypes

	Overall (*n* = 536)		PSP (*n* = 175)		CBD (*n* = 114)		AGD (*n* = 230)		Other 4R (*n* = 67)	
	Females (*n* = 253)	Males (*n* = 283)	Females (*n* = 77)	Males (*n* = 98)	Females (*n* = 50)	Males (*n* = 64)	Females (*n* = 114)	Males (*n* = 116)	Females (*n* = 33)	Males (*n* = 34)
**Age at last visit**	78.2 (11.1)	76.0 (11.0)	77.0 (9.59)	77.1 (9.42)	71.6 (8.55)	67.8 (7.84)	81.8 (10.4)	79.7 (10.2)	77.1 (15.9)	74.6 (14.5)
**Age at death**	80.5 (11.2)	77.9 (11.1)	79.1 (9.93)	78.4 (9.76)	73.1 (8.40)	69.7 (7.79)	84.5 (10.3)	81.8 (10.3)	80.0 (15.6)	77.0 (14.2)
**Years of education**	15.2 (2.85)	16.5 (2.99)	14.9 (2.62)	16.5 (2.93)	16.4 (2.01)	16.5 (3.02)	15.2 (3.06)	16.5 (2.70)	13.6 (2.76)	16.0 (3.75)
**Ethnicity**										
-Hispanic	7 (2.8%)	5 (1.8%)	3 (3.9%)	3 (3.1%)	0	1 (1.6%)	1 (0.9%)	0	4 (12.1%)	1 (2.9%)
-Non-Hispanic	245 (96.8%)	277 (97.9%)	74 (96.1%)	95 (96.9%)	49 (98.0%)	63 (98.4%)	113 (99.1%)	115 (99.1%)	29 (87.9%)	33 (97.1%)
-Not reported	1 (0.4%)	1 (0.4%)	0	0	1 (2%)	0	0	1 (0.9%)	0	0
**Race**										
-American Indian or Alaska Native	0	1 (0.4%)	0	0	0	0	0	0	0	1 (2.9%)
-Asian	6 (2.4%)	4 (1.4%)	2 (2.6%)	2 (2.0%)	2 (4.0%)	1 (1.6%)	2 (1.8%)	2 (1.7%)	1 (3.0%)	0
-Black	9 (3.6%)	6 (2.1%)	2 (2.6%)	0	1 (2%)	0	4 (3.5%)	4 (3.4%)	2 (6.1%)	3 (8.8%)
-Native Hawaiian or Other Pacific Islander	1 (0.4%)	1 (0.4%)	1 (1.3%)	0	0	0	0	1 (0.9%)	0	0
-White	235 (92.9%)	269 (95.1%)	71 (92.2%)	95 (96.9%)	46 (94%)	63 (98.4%)	108 (94.7%)	108 (93.1%)	30 (90.9%)	30 (88.2%)
-Other	1 (0.4%)	0	1 (1.3%)	0	0	0	0	0	0	0
-Not reported	1 (0.4%)	2 (0.7%)	0	1 (1.0%)	1 (2%)	0	0	1 (0.9%)	0	0
**Marital status**										
-Married/domestic partner	122 (48.2%)	241 (85.2%)	38 (49.4%)	83 (84.7%)	39 (78.0%)	58 (90.6%)	45 (39.5%)	96 (82.8%)	11 (33.3%)	29 (85.3%)
-Widowed	90 (35.6%)	21 (7.4%)	26 (33.8%)	9 (9.2%)	4 (8.0%)	1 (1.6%)	48 (42.1%)	11 (9.5%)	18 (54.5%)	2 (5.9%)
-Divorced/separated	31 (12.3%)	17 (6.0%)	12 (15.6%)	6 (6.1%)	5 (10.0%)	4 (6.3%)	14 (12.3%)	7 (6.0%)	3 (9.1%)	1 (2.9%)
-Never married	9 (3.6%)	4 (1.4%)	1 (1.3%)	0	2 (4.0%)	1 (1.6%)	6 (5.3%)	2 (1.7%)	1 (3.0%)	2 (5.9%)
-Not reported	1 (0.4%)	0	0	0	0	0	1 (0.9%)	0	0	0
**Co-participant sex**										
-Female	117 (46.2%)	258 (91.2%)	33 (42.9%)	87 (88.8%)	14 (28.0%)	60 (93.8%)	61 (53.5%)	105 (90.5%)	18 (54.5%)	30 (88.2%)
-Male	131 (51.8%)	20 (7.1%)	41 (53.2%)	9 (9.2%)	36 (72.0%)	4 (6.3%)	52 (45.1%)	9 (7.8%)	14 (42.4%)	3 (8.8%)
-Not reported	6 (2.4%)	5 (1.8%)	3 (3.9%)	2 (2.0%)	0	0	1 (0.9%)	2 (1.7%)	1 (3.0%)	1 (2.9%)

Variables are reported as mean (standard deviation) or count (percentage). PSP: progressive supranuclear palsy, CBD: corticobasal degeneration, AGD: argyrophilic grain disease

#### Overall cohort

The majority identified as non-Hispanic (97.4%) and White (94.0%). Interval between last visit and death was 2.11 (2.29) years. Females were older at last visit (t=−2.39, *p*=.032), death (t=−2.76, *p=*.011), less likely to be married (χ^2^ = 86.4, *p*<.001), and had lower years of education compared to males (t = 5.03, *p*<.001). Most co-participants were females (70%). Males were more likely to have female co-participants, females had a similar percentage of female and male co-participants (χ^2^ = 133, *p*<.001).

#### PSP group

Age at last visit, death, ethnicity, and race did not differ by sex (*p*>.46 for all). Compared to males, females were less likely to be married (χ^2^ = 25.8, *p*<.001) or have female co-participants (χ^2^ = 42.6, *p*<.001); and had lower years of education (t = 3.72, *p*<.001).

#### CBD group

Females were older at last visit than males (t=−2.52, *p*=.046). Age at death, ethnicity, race, education, and marital status did not differ by sex (*p*>.06 for all). Compared to males, females were less likely to have female co-participants (χ^2^ = 53.3, *p*<.001).

#### AGD group

Age at last visit, death, ethnicity, and race did not differ by sex (*p*>.082 for all). Compared to males, females were less likely to be married (χ^2^ = 45.9, *p*<.001) or have female co-participants (χ^2^ = 42.0, *p*<.001). Females had lower years of education than males (t = 3.54, *p*=.002).

#### Other 4R group

Age at last visit, death, ethnicity and race did not differ by sex (*p*>.26 for all). Compared to males, females had lower years of education (t = 2.97, *p*=.009) and were less likely to be married (χ^2^ = 22.2, *p*<.001) or have female co-participants (χ^2^ = 10.1, *p*=.004).

### Sex differences for co-pathologies (Table 2)

**Table 2:** Co-pathologies for the overall 4R cohort and subtypes

	Overall (*n* = 536)		PSP (*n* = 175)		CBD (*n* = 114)		AGD (*n* = 230)		Other 4R (*n* = 67)	
	Females (*n* = 253)	Males (*n* = 283)	Females (*n* = 77)	Males (*n* = 98)	Females (*n* = 50)	Males (*n* = 64)	Females (*n* = 114)	Males (*n* = 116)	Females (*n* = 33)	Males (*n* = 34)
**FTLD-4R-tauopathy**										
-PSP	77 (30.4%)	98 (34.6%)	77 (100%)	98 (100%)	1 (2.0%)	7 (10.9%)	6 (5.3%)	10 (8.6%)	2 (6.1%)	0
-CBD	50 (19.8%)	64 (22.6%)	1 (1.3%)	7 (7.1%)	50 (100%)	64 (100%)	9 (7.9%)	8 (6.9%)	0	0
-AGD	114 (45.1%)	116 (41.0%)	6 (7.8%)	10 (10.2%)	9 (18.0%)	8 (12.5%)	114 (100%)	116 (100%)	4 (12.1%)	4 (11.8%)
-Other 4R-tauopathy	33 (13.0%)	34 (12.0%)	2 (2.6%)	0	0	0	4 (3.5%)	4 (3.4%)	33 (100%)	34 (100%)
**FTLD-3R-tauopathy**										
-Pick’s disease	1 (0.4%)	2 (0.7%)	0	0	0	0	1 (0.9%)	1 (0.9%)	0	1 (2.9%)
-Other 3R-tauopathy including *MAPT* mutation 3R-tauopathy	0	1 (0.4%)	0	1 (1.0%)	0	0	0	0	0	0
-Chronic traumatic encephalopathy	1 (0.4%)	8 (2.8%)	0	4 (4.1%)	0	0	0	2 (1.7%)	1 (3.0%)	2 (5.9%)
-ALS/parkinsonism-dementia	1 (0.4%)	1 (0.4%)	1 (1.3%)	0	0	0	0	0	0	0
-Tangle dominant disease	9 (3.6%)	10 (3.5%)	2 (2.6%)	2 (2.0%)	1 (2.0%)	1 (1.6%)	7 (6.1%)	7 (6.0%)	0	1 (2.9%)
-Other 3R + 4R tauopathy	16 (6.3%)	18 (6.4%)	2 (2.6%)	2 (2.0%)	1 (2.0%)	1 (1.6%)	12 (10.5%)	13 (11.2%)	3 (9.1%)	3 (8.8%)
**FTLD with TDP-43**	16 (6.3%)	12 (4.2%)	4 (5.2%)	2 (2.0%)	2 (4.0%)	4 (6.3%)	11 (9.6%)	5 (4.3%)	1 (3.0%)	1 (2.9%)
**ALS/motor neuron disease**	2 (0.8%)	3 (1.1%)	1 (1.3%)	2 (2.0%)	0	1 (1.6%)	1 (0.9%)	1 (0.9%)	0	0
**Other FTLD**	3 (1.2%)	1 (0.4%)	0	0	0	0	0	1 (0.9%)	0	0
**ARTAG presence**	59 (23.3%)	60 (21.2%)	15 (19.5%)	19 (19.4%)	9 (18.0%)	8 (12.5%)	36 (31.6%)	36 (31.0%)	3 (9.1%)	4 (1.8%)
**ARTAG severity**	1.22 (0.46)	1.30 (0.60)	1.17 (0.39)	1.56 (0.81)	1 (0)	1.29 (0.76)	1.28 (0.51)	1.20 (0.41)	1 (0)	1 (0)
**Ischemic/hemorrhagic/vascular pathology**	245 (96.8%)	271 (95.8%)	73 (94.8%)	95 (96.9%)	48 (96.0%)	63 (98.4%)	114 (100%)	112 (96.6%)	31 (93.9%)	29 (85.3%)
**Cerebral amyloid angiopathy**	0.78 (0.88)	0.80 (0.95)	0.51 (0.70)	0.61 (0.95)	0.45 (0.79)	0.41 (0.78)	1.08 (0.92)	1.13 (0.92)	0.83 (0.83)	0.82 (1.03)
**Hippocampal sclerosis**	29 (11.5%)	31 (11.0%)	6 (7.8%)	9 (9.2%)	3 (6.0%)	4 (6.3%)	18 (15.8%)	14 (12.1%)	5 (15.2%)	4 (11.8%)
**Thal amyloid β plaque score**	2.52 (1.89)	2.44 (1.88)	2.00 (1.75)	1.97 (1.72)	1.47 (1.49)	1.56 (1.51)	3.12 (1.84)	3.07 (1.96)	2.63 (1.88)	2.50 (1.89)
**Braak neurofibrillary tangle stage**	3.17 (1.82)	3.05 (1.95)	2.70 (1.56)	2.51 (1.79)	1.69 (1.60)	1.68 (1.59)	3.77 (1.60)	3.76 (1.71)	3.38 (2.16)	3.31 (2.35)
**CERAD neuritic plaque score**	1.26 (1.23)	1.16 (2.22)	0.99 (1.16)	0.79 (1.05)	0.89 (1.15)	0.78 (1.08)	1.47 (1.22)	1.54 (1.24)	1.50 (1.28)	1.12 (1.30)
**CERAD diffuse plaque score**	1.91 (1.19)	1.80 (1.27)	1.65 (1.20)	1.48 (1.28)	1.41 (1.19)	1.30 (1.16)	2.17 (1.08)	2.16 (1.23)	2.03 (1.24)	1.77 (1.28)
**Lewy body pathology stage**										
-None	190 (75.1%)	192 (67.8%)	60 (77.9%)	77 (78.6%)	46 (92.0%)	55 (85.9%)	76 (66.7%)	61 (52.6%)	26 (78.8%)	24 (70.6%)
-Olfactory bulb	2 (0.8%)	3 (1.1%)	2 (2.6%)	0	0	0	1 (0.9%)	2 (1.7%)	0	1 (2.9%)
-Amygdala	18 (7.1%)	19 (6.7%)	4 (5.2%)	5 (5.1%)	1 (2.0%)	2 (3.1%)	10 (8.8%)	8 (6.9%)	4 (12.1%)	4 (11.8%)
-Brainstem	15 (5.9%)	19 (6.7%)	5 (6.5%)	4 (4.1%)	2 (4.0%)	4 (6.3%)	7 (6.1%)	12 (10.3%)	1 (3.0%)	1 (2.9%)
-Limbic	13 (5.1%)	24 (8.5%)	5 (6.5%)	6 (6.1%)	0	3 (4.7%)	7 (6.1%)	15 (12.9%)	1 (3.0%)	2 (5.9%)
-Neocortex	14 (5.5%)	25 (8.8%)	1 (1.3%)	6 (6.1%)	1 (2.0%)	0	13 (11.4%)	17 (14.7%)	1 (3.0%)	2 (5.9%)
**Substantia nigra neuron loss**	1.09 (1.03)	1.36 (1.08)	1.16 (1.18)	1.44 (1.11)	1.69 (1.08)	1.97 (1.03)	0.77 (0.75)	1.01 (0.96)	1.35 (1.05)	1.64 (0.99)

Variables are reported as mean (standard deviation) or count (percentage). PSP: progressive supranuclear palsy, CBD: corticobasal degeneration, AGD: argyrophilic grain disease, FTLD: frontotemporal lobar degeneration, ALS: amyotrophic lateral sclerosis, ARTAG: aging-related tau astrogliopathy

#### Overall cohort

**Fig. 2:**
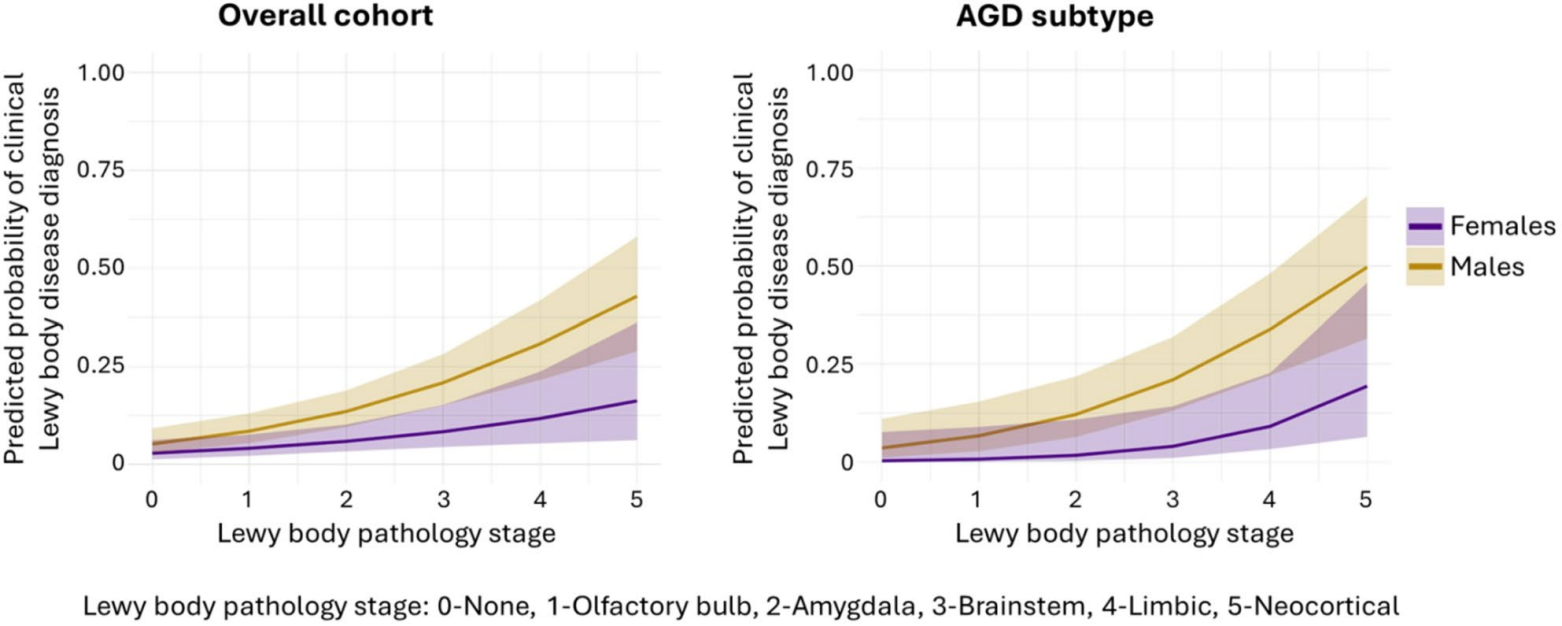
Association between Lewy body pathology staging and clinical diagnosis of Lewy body disease in the overall cohort and argyrophilic grain disease (AGD) subtype adjusted for age at last visit

In models adjusted for age at death, females were less likely to have CTE (Wald χ^2^ = 3.91, *p*=.048) and had lower Lewy body pathology staging (Wald’s χ^2^ = 8.04, *p*=.005). Other co-pathology variables did not differ by sex (*p*>.052 for all). In models adjusted for age at last visit, higher Lewy body pathology was associated with a higher likelihood LBD clinical diagnosis more for males than females (Wald χ^2^ = 47.5, *p*<.001; odds ratio[OR] = 1.34 for females vs. OR = 1.80 for males; Fig. 2).

#### PSP, CBD, Other 4R groups

There were no sex differences for co-pathologies (*p*>.091 for all).

#### AGD group

Females had lower Lewy body pathology staging than males (Wald’s χ^2^ = 5.85, *p*=.016), without other co-pathology differences (*p*>.079 for all). Higher Lewy body pathology stage was associated with a higher likelihood for LBD clinical diagnosis more for males than females (Wald χ^2^ = 30.0, *p*<.001; OR = 1.61 for females vs. OR = 2.27 for males; Fig. 2).

### Sex differences for clinical profiles (Table 3; Fig. 3)

**Table 3:** Clinical features for the cohort

	Overall (*n* = 536)		PSP (*n* = 175)		CBD (*n* = 114)		AGD (*n* = 230)		Other 4R (*n* = 67)	
	Females (*n* = 253)	Males (*n* = 283)	Females (*n* = 77)	Males (*n* = 98)	Females (*n* = 50)	Males (*n* = 64)	Females (*n* = 114)	Males (*n* = 116)	Females (*n* = 33)	Males (*n* = 34)
**Predominant domain first recognized as changed**										
-Cognition	147 (58.1%)	195 (68.9%)	32 (41.6%)	67 (68.4%)	30 (60.0%)	40 (62.5%)	76 (66.7%)	87 (75.0%)	21 (63.6%)	22 (64.7%)
-Behavior	31 (12.3%)	27 (9.5%)	8 (10.4%)	4 (4.1%)	11 (22.0%)	14 (21.9%)	10 (8.8%)	7 (6.0%)	6 (18.2%)	5 (14.7%)
-Motor	41 (16.2%)	40 (14.1%)	30 (39.0%)	22 (22.4%)	7 (14.0%)	9 (14.1%)	5 (4.4%)	8 (6.9%)	1 (3.0%)	5 (14.7%)
**Predominant cognitive domain first recognized as changed**										
-Memory	99 (39.1%)	108 (38.2%)	24 (31.2%)	30 (30.6%)	9 (18.0%)	7 (10.9%)	60 (52.6%)	63 (54.3%)	16 (48.5%)	17 (50.0%)
-Orientation	0	0	0	0	0	0	0	0	0	0
-Executive	41 (16.2%)	68 (24.0%)	21 (27.3%)	27 (27.6%)	12 (24.0%)	25 (39.1%)	9 (7.9%)	13 (11.2%)	3 (9.1%)	8 (23.5%)
-Language	48 (19.0%)	61 (21.6%)	16 (20.8%)	24 (24.5%)	22 (44.0%)	24 (37.5%)	9 (7.9%)	21 (18.1%)	3 (9.1%)	4 (11.8%)
-Visuospatial	4 (1.6%)	7 (2.5%)	0	1 (1.0%)	3 (6.0%)	5 (7.8%)	1 (0.9%)	1 (0.9%)	0	0
-Attention	7 (2.8%)	4 (1.4%)	3 (3.9%)	2 (2.0%)	1 (2.0%)	0	5 (4.4%)	1 (0.9%)	0	1 (2.9%)
-Cognitive fluctuation	1 (0.4%)	0	0	0	0	0	1 (0.9%)	0	0	0
**Dementia**	190 (75.1%)	242 (85.8%)	60 (77.9%)	81 (82.7%)	42 (84.0%)	62 (96.9%)	79 (69.3%)	93 (80.2%)	27 (81.8%)	32 (94.1%)
**CDR-Sum of Boxes**	8.07 (6.44)	8.95 (5.67)	7.29 (6.17)	9.45 (5.68)	10.8 (6.25)	11.2 (5.77)	7.48 (6.61)	7.89 (5.80)	8.42 (5.99)	8.85 (5.24)
**Age at cognitive decline onset**	69.8 (11.2)	67.5 (10.8)	68.7 (10.2)	68.3 (8.96)	65.3 (6.25)	61.9 (7.33)	73.1 (11.3)	70.5 (11.1)	67.7 (14.1)	65.2 (14.9)
**Memory impairment**	185 (73.1%)	246 (86.9%)	53 (68.8%)	83 (84.7%)	39 (78.0%)	58 (90.6%)	84 (73.7%)	100 (86.2%)	27 (81.8%)	31 (91.2%)
**Orientation impairment**	109 (43.1%)	144 (50.9%)	24 (31.2%)	50 (51.0%)	23 (46.0%)	32 (50.0%)	55 (48.2%)	54 (46.6%)	16 (48.5%)	25 (73.5%)
**Judgment impairment**	196 (77.5%)	246 (86.9%)	62 (80.5%)	89 (90.8%)	46 (92.0%)	61 (95.3%)	78 (68.4%)	91 (78.4%)	27 (81.8%)	30 (88.2%)
**Language impairment**	150 (59.3%)	188 (66.4%)	53 (68.8%)	72 (73.5%)	41 (82.0%)	51 (81.3%)	49 (43.0%)	66 (56.9%)	18 (54.5%)	22 (64.7%)
**Visuospatial impairment**	79 (31.2%)	107 (37.8%)	26 (33.8%)	41 (41.8%)	24 (48.0%)	31 (48.4%)	27 (23.7%)	32 (27.6%)	9 (27.3%)	11 (32.4%)
**Attention impairment**	117 (46.2%)	154 (54.4%)	35 (45.5%)	60 (61.2%)	24 (48.0%)	41 (64.1%)	50 (43.9%)	53 (45.7%)	16 (48.5%)	17 (50.0%)
**Cognitive fluctuations**	12 (4.7%)	33 (11.7%)	4 (5.2%)	13 (13.3%)	3 (6.0%)	5 (7.8%)	4 (3.5%)	14 (21.1%)	2 (6.1%)	2 (5.9%)
**Predominant behavior symptom first recognized**										
-Apathy	55 (21.7%)	73 (25.8%)	24 (31.2%)	26 (26.5%)	12 (24.0%)	31 (48.4%)	17 (14.9%)	21 (18.1%)	6 (18.2%)	5 (14.7%)
-Depression	43 (17.0%)	22 (7.8%)	15 (19.5%)	11 (11.2%)	6 (12.0%)	7 (10.9%)	23 (20.2%)	7 (6.0%)	2 (6.1%)	1 (2.9%)
-Psychosis	3 (1.2%)	4 (1.4%)	1 (1.3%)	2 (2.0%)	0	1 (1.6%)	2 (1.8%)	1 (0.9%)	1 (3.0%)	1 (2.9%)
-Disinhibition	12 (4.7%)	13 (4.6%)	2 (2.6%)	3 (3.1%)	5 (10.0%)	2 (3.1%)	5 (4.4%)	6 (5.2%)	1 (3.0%)	4 (11.8%)
-Irritability	11 (4.3%)	31 (11.0%)	4 (5.2%)	9 (9.2%)	2 (4.0%)	6 (9.4%)	3 (2.6%)	11 (9.5%)	3 (9.1%)	6 (17.6%)
-Agitation	2 (0.8%)	8 (2.8%)	1 (1.3%)	4 (4.1%)	1 (2.0%)	2 (3.1%)	1 (0.9%)	3 (2.6%)	0	0
-Personality change	17 (6.7%)	24 (8.5%)	6 (7.8%)	6 (6.1%)	8 (16.0%)	8 (12.5%)	1 (0.9%)	4 (3.4%)	3 (9.1%)	6 (17.6%)
-RBD	1 (0.4%)	8 (2.8%)	0	1 (1.0%)	0	0	1 (0.9%)	6 (5.2%)	0	1 (2.9%)
-Anxiety	8 (3.2%)	12 (4.2%)	1 (1.3%)	5 (5.1%)	3 (6.0%)	1 (1.6%)	4 (3.5%)	4 (3.4%)	0	3 (8.8%)
**NPI-Q**	5.62 (4.95)	6.44 (5.46)	5.65 (4.38)	5.17 (3.98)	7.06 (4.97)	8.06 (5.71)	4.42 (4.68)	6.16 (5.99)	7.13 (5.67)	6.21 (5.31)
**Age at behavioral symptom onset**	71.7 (10.9)	67.1 (11.7)	70.8 (8.38)	69.3 (9.80)	68.2 (7.72)	63.7 (8.00)	76.3 (11.1)	69.9 (11.2)	63.1 (15.3)	65.0 (18.3)
**Apathy**	110 (43.5%)	156 (55.1%)	42 (54.5%)	55 (56.1%)	29 (58.0%)	57 (89.1%)	38 (33.3%)	45 (38.8%)	11 (33.3%)	16 (47.1%)
**Depression**	74 (29.2%)	76 (26.9%)	30 (39.0%)	35 (35.7%)	13 (26.0%)	19 (29.7%)	30 (26.3%)	20 (17.2%)	6 (18.2%)	6 (17.6%)
**Hallucinations**	13 (5.1%)	10 (3.5%)	2 (2.6%)	2 (2.0%)	2 (4.0%)	1 (1.6%)	9 (7.9%)	8 (6.9%)	1 (3.0%)	2 (5.9%)
**Delusions**	22 (8.7%)	27 (9.5%)	5 (6.5%)	7 (7.1%)	4 (8.0%)	5 (7.8%)	11 (9.6%)	14 (12.1%)	5 (15.2%)	2 (5.9%)
**Disinhibition**	50 (19.8%)	70 (24.7%)	11 (14.3%)	16 (16.3%)	19 (38.0%)	26 (40.6%)	15 (13.2%)	22 (19.0%)	9 (27.3%)	10 (29.4%)
**Irritability**	64 (25.3%)	88 (31.1%)	23 (29.9%)	26 (26.5%)	15 (30.0%)	31 (48.4%)	22 (19.2%)	23 (19.8%)	10 (30.3%)	11 (32.4%)
**Agitation**	44 (17.4%)	60 (21.2%)	10 (13.0%)	22 (22.4%)	16 (32.0%)	22 (34.4%)	17 (14.9%)	18 (15.5%)	5 (15.2%)	5 (14.7%)
**Personality change**	49 (19.4%)	71 (25.1%)	13 (16.9%)	22 (22.4%)	19 (38.0%)	30 (46.9%)	14 (12.3%)	14 (12.1%)	9 (27.3%)	12 (35.3%)
**RBD**	4 (1.6%)	13 (4.6%)	3 (3.9%)	3 (3.1%)	1 (2.0%)	2 (3.1%)	1 (0.9%)	8 (6.9%)	0	1 (2.9%)
**Anxiety**	43 (17.0%)	62 (21.9%)	15 (19.5%)	21 (21.4%)	9 (18.0%)	20 (31.3%)	16 (14.0%)	19 (16.4%)	5 (15.2%)	8 (23.5%)
**Predominant motor symptom first recognized**										
-Gait disorder	39 (15.4%)	64 (22.6%)	19 (24.7%)	27 (27.6%)	6 (12.0%)	14 (21.9%)	14 (12.3%)	24 (20.7%)	2 (6.1%)	6 (17.6%)
-Falls	39 (15.4%)	27 (9.5%)	24 (31.2%)	19 (19.4%)	9 (18.0%)	6 (9.4%)	7 (6.1%)	3 (2.6%)	2 (6.1%)	0
-Tremors	14 (5.5%)	32 (11.3%)	4 (5.2%)	9 (9.2%)	1 (2.0%)	6 (9.4%)	7 (6.1%)	14 (12.1%)	2 (6.1%)	5 (14.7%)
-Slowness	39 (15.4%)	63 (22.3%)	9 (11.7%)	27 (27.6%)	18 (36.0%)	21 (32.8%)	13 (11.4%)	19 (16.4%)	2 (6.1%)	7 (20.6%)
**Age at motor symptom onset**	70.9 (10.9)	70.4 (10.5)	69.9 (8.73)	72.0 (8.71)	68.7 (8.11)	64.6 (7.64)	75.6 (10.6)	74.9 (10.3)	56.5 (19.2)	65.8 (14.8)
**Gait disorder**	114 (45.1%)	161 (56.9%)	55 (71.4%)	71 (72.4%)	29 (58.0%)	44 (68.8%)	32 (28.1%)	50 (43.1%)	6 (18.2%)	17 (50.0%)
**Falls**	83 (32.8%)	123 (43.5%)	45 (58.4%)	67 (68.4%)	21 (42.0%)	32 (50.0%)	19 (16.7%)	30 (25.9%)	5 (15.2%)	10 (29.4%)
**Tremors**	52 (20.6%)	87 (30.7%)	20 (26.0%)	29 (29.6%)	12 (24.0%)	27 (42.2%)	18 (15.8%)	29 (25.0%)	4 (12.1%)	12 (35.3%)
**Slowness**	112 (44.3%)	152 (53.7%)	52 (67.5%)	70 (71.4%)	32 (64.0%)	41 (64.1%)	27 (23.7%)	42 (36.2%)	7 (21.2%)	16 (47.1%)

#### Overall cohort

For the overall course of decline of the predominant cognitive/behavioral/motor syndrome, the majority of the cohort (97.7%) experienced gradual decline without sex differences (98.6% for females vs. 96.9% for males, χ^2^ = 4.93, *p*=.29). For both females and males, memory decline was the most common cognitive symptom; apathy was the most common behavioral symptom; cognition was the most affected domain out of cognition, behavior, and motor options. Overall, 80.6% had dementia, 45.6% had FTD clinical diagnosis, 40.7% had AD clinical diagnosis, 8.8% had LBD clinical diagnosis.

Percentages of the predominant cognitive, behavioral, and motor symptoms first recognized differed by sex (χ^2^ = 16.6, *p*=.020; χ^2^ = 28.5, *p*=.002; χ^2^ = 23.0, *p*<.001). Adjusted for age at last visit and years of education, females were less likely than males to have dementia (Wald χ^2^ = 31.8, *p*<.001); cognitive fluctuations (Wald χ^2^ = 6.57, *p*=.010); decline in memory (Wald χ^2^ = 14.6, *p*<.001), judgment (Wald χ^2^ = 6.00, *p*=.014); were older at cognitive decline onset (Wald χ^2^ = 5.15, *p*=.023). Adjusted for age at last visit, females were less likely than males to have apathy (Wald χ^2^ = 4.29, *p*=.038); gait disorder (Wald χ^2^ = 4.91, *p*=.027); falls (Wald χ^2^ = 4.14, *p*=.042); tremor (Wald χ^2^ = 6.05, *p*=.014); were older at behavior change onset (Wald χ^2^ = 5.00, *p*=.025). Compared to males, females were less likely to have a clinical diagnosis of LBD (Wald χ^2^ = 10.4, *p*=.001). Other clinical features and diagnoses did not differ by sex (*p*>.059 for all). Sex differences for cognitive decline and behavioral symptom onset ages were not age dependent (*p*=.13, *p*=.86, respectively).

#### PSP group

PSP was the most common clinical diagnosis (41.1%) without sex differences. Falls were the most common first motor symptom for females; gait disorder and slowness were the most common first motor symptoms for males (χ^2^ = 11.2, *p*=.024). Both cognition and motor were the first affected domain for females; majority of males had cognition as the first affected domain (χ^2^ = 11.9, *p*=.003). Females were less likely than males to have decline in memory (Wald χ^2^ = 8.09, *p*=.044); orientation (Wald χ^2^ = 6.14, *p*=.013); judgment (Wald χ^2^ = 5.98, *p*=.014); attention (Wald χ^2^ = 5.75, *p*=.017); had less severe dementia (Wald χ^2^ = 8.25, *p*=.004). Other clinical features and diagnoses did not differ by sex (*p*>.065 for all). Sex difference for dementia severity was not age dependent (*p*=.10).

#### CBD group

Unspecified FTD was the most common clinical diagnosis (36.0%), followed by CBS (24.6%) without sex differences. Females were less likely than males to have apathy (Wald χ^2^ = 10.7, *p*=.001); tremors (Wald χ^2^ = 4.18, *p*=.041); were older at behavioral symptom onset (Wald χ^2^ = 6.29, *p*=.012). There were no other sex differences (*p*>.053 for all). Sex difference for behavioral symptom onset was not age dependent (*p*=.98).

#### AGD group

AD was the most common clinical diagnosis (57.0%) without sex differences. The most common first behavioral symptom was depression for females; apathy for males (χ^2^ = 20.7, *p*=.024). Gait disorder was more common as the first motor symptom for males than females (χ^2^ = 11.9, *p*=.003). Females were less likely than males to have cognitive fluctuations (Wald χ^2^ = 4.71, *p*=.030); gait disorder (Wald χ^2^ = 4.79, *p*=.029); clinical diagnosis of LBD (Wald χ^2^ = 9.27, *p*=.002); were more likely to have depression (Wald χ^2^ = 4.05, *p*=.044); had behavioral symptom onset at an older age and less severe behavioral symptoms (Wald χ^2^ = 4.53, *p*=.033, Wald χ^2^ = 4.45, *p*=.035, respectively). There were no other sex differences (*p*>.051 for all). Sex differences for behavioral symptom onset age and severity were not dependent on age (*p*=.99, *p*=.55, respectively).

#### Other 4R group

AD was the most common clinical diagnosis (47.8%) without sex differences. Females were older at cognitive decline onset (Wald χ^2^ = 4.37, *p*=.037); less likely to have gait disorder (Wald χ^2^ = 6.40, *p*=.011), tremors (Wald χ^2^ = 4.50, *p*=.034), and slowness (Wald χ^2^ = 4.49, *p*=.034). There were no other sex differences (*p*>.075 for all). Sex difference for cognitive decline onset age was not dependent on age (*p*=.82).

**Fig. 3:**
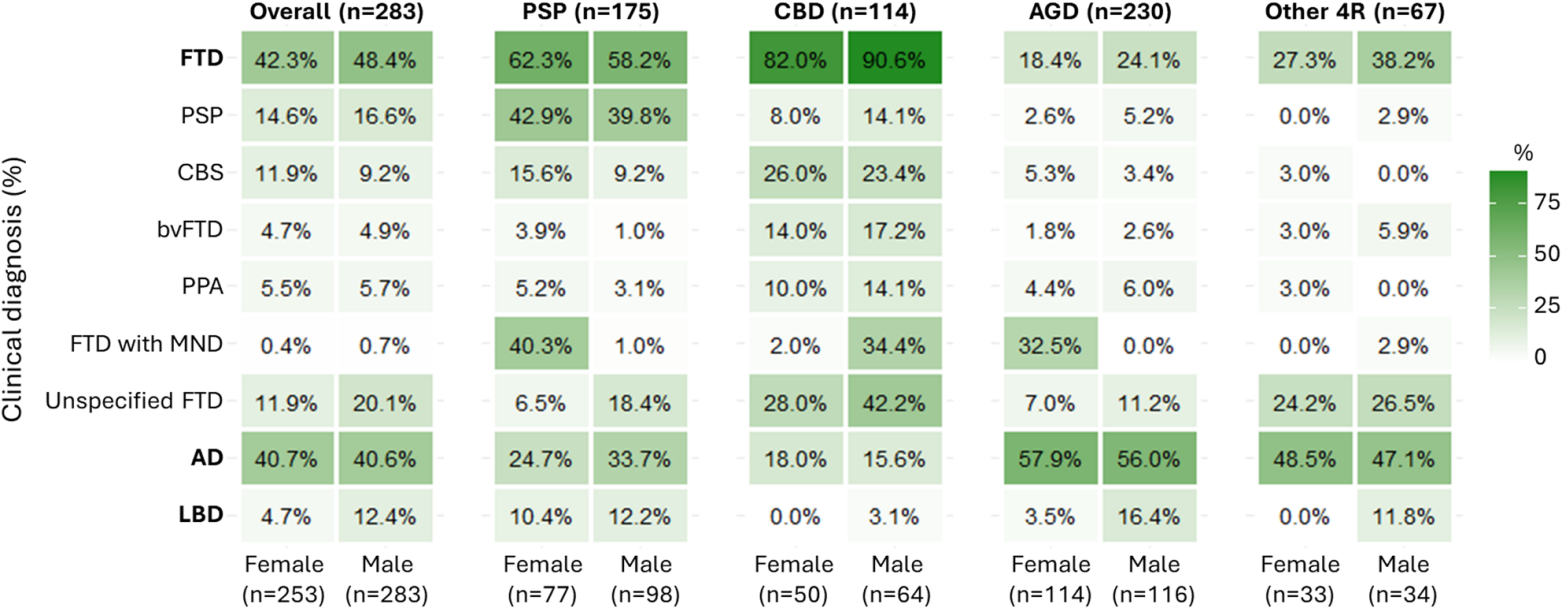
Clinical diagnoses across overall cohort, progressive supranuclear palsy (PSP), corticobasal degeneration (CBD), argyrophilic grain disease (AGD), and other 4R groups. FTD: frontotemporal dementia used as an umbrella term, PSP: progressive supranuclear palsy, CBS: corticobasal syndrome, bvFTD: behavioral frontotemporal dementia, PPA: primary progressive aphasia, MND: motor neuron disease, AD: Alzheimer’s disease, LBD: Lewy body disease

## Discussion

Using a pathologically defined cohort of 4R-tauopathy, we found similar ratios of females and males across the different 4R-tauopathy subtypes without prominent sex differences for co-pathologies, except for Lewy body pathology. Other than vascular pathology, co-pathologies were not frequent across the groups. Females tend to outlive males despite worse overall health and higher rates of disability [28], and this sex difference in survival was preserved in our cohort. To account for this survival bias and potential inclusion bias of females with milder diseases over males with more advanced and severe diseases, we included age as a covariate in our models and also performed sensitivity analyses with age as a moderating variable. These sensitivity analyses revealed the relatively older onset age and less severe symptoms for females compared to males were not necessarily age dependent. Females being less likely to be married/partnered and more likely to be widowed in our cohort couples this survival bias. Most caregivers for people with neurodegenerative disorders including AD, FTD, and LBD, are women [29–31]. Similarly, most co-participants in our cohort were females despite a similar sex distribution across the pathology groups.

Both females and males had a limited clinical diagnostic accuracy during life emphasizing the shortcomings of clinical criteria and the need for biomarkers during life. The participants with pathology data represent older data in the NACC, and the diagnostic criteria for both PSP [32] and CBD [33] have been updated over time providing a better diagnostic accuracy. However, our findings, in line with the literature [9], show that these pathologies are associated with a range of clinical profiles linked to pathologies such as AD, LBD, and other tauopathies. In recognition of these clinical profiles, caregiver report plays an important role for a range of symptoms. Caregiver gender can impact the report of symptoms as well as severity. For instance, women caregivers are more likely to notice subtle change in cognition, behavior, and daily functioning in the early stages of dementia [34]. The higher percentage of females as co-participants in our cohort likely affected our findings. These emphasize the need to incorporate biomarkers for better clinical diagnostic accuracy. With focus on the more affordable and less invasive biomarkers, 4R tau seed amplification assays in skin have been providing promising results for the clinical diagnosis of PSP [35, 36]. As co-pathologies can occur in 4R-tauopathies, use of different biomarkers in combination can provide a better insight into the multiple neurodegenerative pathologies that may be impacting the individual.

Mahale and colleagues reported younger age at onset and more frequent apathy, executive dysfunction, and hyperreflexia, as well as faster progression to severe dysarthria, dysphagia, and cognitive impairment for females with PSP [11]. Despite more frequent PSP-parkinsonism subtype and faster progression to wheelchair dependency for males, females were more likely to experience falls within the first year [11]. Our findings also revealed falls as the most common first motor symptom for females, compared to gait disorder as a more common first motor symptom for males. Since both patient and caregiver sex and gender impact reporting of symptoms [34], changes in gait for females may remain underrecognized prior to falls. In clinically defined PSP cohorts, a study noted faster disease progression for females [10], and another study showed younger age at diagnosis for females with longer survival after diagnosis [37]. In contrast, another study reported no sex differences in disease onset age, symptoms, or progression [13]. Similarly, we did not find a difference in clinical diagnoses, onset age and age at death by sex. Despite studies suggesting worse cognition for females [11, 12] or lack of sex differences for cognition [14], we found less severe dementia and lower likelihood of attention, executive, and memory impairment for females than males. As only 41% of people with PSP pathology had clinical PSP diagnosis in our cohort, it is not surprising that our findings do not completely align with previous findings. However, studies showing lower PSP risk for females with history of estrogen replacement therapy [38], and female-specific benefit with davunetide that enhances tau-microtubule interaction to protect against tauopathy and neurodegeneration [10], support the consideration of sex in PSP for risk, diagnosis, and treatment.

We found a similar prevalence and co-pathology profile for females and males in the CBD group. Clinical diagnosis of FTD was more common than AD and LBD in the CBD group in line with previous literature [33]. Although CBS is typically the most common clinical profile for CBD, unspecified FTD clinical profile was more frequent than CBS. This can be due to participants with neuropathological assessments in the NACC having been clinically assessed prior to the 2013 Armstrong diagnostic criteria for CBD outlining the probable and possible CBS clinical profiles [33]. It also supports the utility of the 4R-tauopathy diagnostic criteria for better sensitivity [6]. Tremors are less common than other movement symptoms in CBD [33]. Accordingly, we noted tremors as the least common movement symptom for both females and males, with females showing even lower incidence than males. Apathy is common in CBD, and other neurodegenerative diseases including PSP, AD, FTD, and LBD [32, 39–41]. There is a male predominance for apathy in AD, FTD, and dementia with Lewy bodies [40], which we also noted in our CBD cohort with older age at behavioral symptom onset for females. In an FTD cohort, females were less likely to have apathy than males despite similar levels of frontotemporal atrophy [42]. Thus, the sex difference for apathy in our cohort can stem from gender-related differences in co-participant report of symptoms and a potential resilience in females with CBD. While we did not find a sex difference for survival in CBD, Aiba and colleagues reported longer survival for females than males without sex differences for clinical profile [43]. Our findings likely differ due to their CBD cohort consisting of people with primarily CBS and PSP clinical diagnoses with movement symptoms, whereas our NACC cohort includes ADRCs collecting data to advance AD and related dementia research.

Behavioral symptoms are common in AGD with depression being one of the most common [19, 44]. Compared to males, females were more likely to have depression but had overall less severe behavioral symptoms with an older age at onset. Interestingly, females were less likely to have clinical LBD diagnosis than males, including lower likelihood of cognitive fluctuations and gait disorder, coupled with a lower Lewy body pathology staging. Previous findings support a lower prevalence and less severe Lewy body pathology in females compared to males [45, 46]. Additionally, females have a lower likelihood of LBD clinical profile than males despite similar or more Lewy body pathology burden [23, 47]. Our findings outline that Lewy body pathology frequency, and clinical associations differ by sex in the overall 4R-tauopathy group and the AGD subtype, suggesting a potential resilience against Lewy body pathology in females. Tau and alpha-synuclein pathologies can enhance the aggregation of one another and have synergistic neurodegenerative effects [48]. In AD, alpha-synuclein co-pathology is associated with faster tau accumulation for females than males [49]. Thus, the interaction of alpha-synuclein and tau for clinical outcomes may differ by sex and depend on the predominant or initial pathology, as well as the type of tauopathy.

In the overall cohort, CTE prevalence differed by sex. Traumatic brain injury (TBI) is associated with CTE and Lewy body pathology [50]. Traditionally, males have higher TBI prevalence [51], however, recent studies have suggested TBI incidence can be higher for females in the age group of > 65 [52]. Outcomes of TBI can differ by sex; females experience more and worse symptoms and more structural changes on magnetic resonance imaging in brain regions that are associated with tau pathology in CTE [53]. Thus, sex differences can occur in the prevalence of pathologies with changes in gender roles in daily life. Sex differences in clinicopathological associations should not be disregarded for tau and Lewy body pathologies.

Other 4R subtype in our cohort included different 4R-tauopathies and had a small number of participants. Thus, the findings from this subtype are more exploratory and should be interpreted cautiously. Older age at cognitive decline onset for females in the Other 4R subtype is consistent with a previous review reporting females 6 years older at symptom onset than males with GGT [15]. Buciuc and colleagues noted eye movement abnormalities, parkinsonism, and behavioral changes as the most common symptoms for GGT [54]. While motor symptoms were observed for half of the males in the Other 4R subtype, females were significantly less likely to experience motor symptoms. However, we were unable to ascertain whether majority of this subtype consisted of people with GGT or another type of 4R-tauopathy.

### Perspectives and significance

Given the limited clinical diagnostic accuracy in 4R-tauopathies, as also demonstrated by our findings regarding the concordance between clinical diagnosis and pathological diagnosis, use of a large pathologically defined cohort is a strength of our study. However, NACC includes participants recruited through ADRCs which leads to a bias for the demographics and clinical diagnosis. This clinical diagnosis bias can be particularly important for PSP and CBD, given the movement disorders associated with both pathologies that may not necessarily lead to a referral to an ADRC. Clinical diagnoses are based on the available diagnostic criteria at the time, which have been updated throughout the years [25, 26]. Clinician reports of symptoms are based on interviews with individual and co-participant, clinical and cognitive exams [26, 27]. While validated scales are included in NACC, scales may not be administered to all participants, and some reports may be subjective. Comorbidities and treatments can impact the presence and severity of symptoms, which were not assessed in our analyses. Age is a major risk factor for neurodegeneration and the sex difference for age at death may have impacted our findings. Although we conducted sensitivity analyses to account for this sex difference in survival, our small sample size may have lacked power to detect age interactions. Despite these limitations, our analyses highlight the sex differences in neurodegenerative diseases expanding beyond the more prevalent diseases with more extensive research such as AD and LBD.

## Conclusions

The prevalence of co-existing neurodegenerative pathologies in this 4R-tau pathological cohort was similar between sexes, with only Lewy body pathology impacting males more. Despite similar pathologies and clinical diagnoses, age at onset and the specific type and relative predominance of cognitive, behavioral, and motor symptoms can differ by sex in 4R-tauopathies. Females had a lower risk of cognitive decline, apathy, and motor symptoms, and experienced cognitive and behavioral changes at an older age than males, suggesting a potential female advantage in 4R-tauopathies.

## Data Availability

The dataset supporting the conclusions of this article is available upon request from the NACC in https://naccdata.org.
